# Conditional deletion of AP-2β in mouse cranial neural crest results in anterior segment dysgenesis and early-onset glaucoma

**DOI:** 10.1242/dmm.025262

**Published:** 2016-08-01

**Authors:** Vanessa B. Martino, Thomas Sabljic, Paula Deschamps, Rebecca M. Green, Monica Akula, Erica Peacock, Alexander Ball, Trevor Williams, Judith A. West-Mays

**Affiliations:** 1 Department of Pathology and Molecular Medicine, McMaster University Health Science Centre, Room 4N65, 1200 Main St. West, Hamilton, Ontario, CanadaL8N 3Z5; 2 Department of Craniofacial Biology, University of Colorado Denver, Anschutz Medical Campus, Mailstop 8120, RC-1 South Building, 11th Floor, Room 111, 12801 East 17th Ave. P.O., Aurora, CO 80045, USA; 3 Department of Cell and Developmental Biology, University of Colorado Denver, Anschutz Medical Campus, Mailstop 8120, RC-1 South Building, 11th Floor, Room 111, 12801 East 17th Ave. P.O., Aurora, CO 80045, USA

**Keywords:** Anterior segment dysgenesis, Glaucoma, AP-2β

## Abstract

Anterior segment dysgenesis (ASD) encompasses a group of developmental disorders in which a closed angle phenotype in the anterior chamber of the eye can occur and 50% of patients develop glaucoma. Many ASDs are thought to involve an inappropriate patterning and migration of the periocular mesenchyme (POM), which is derived from cranial neural crest cells (NCCs) and mesoderm. Although, the mechanism of this disruption is not well understood, a number of transcriptional regulatory molecules have previously been implicated in ASDs. Here, we investigate the function of the transcription factor AP-2β, encoded by *Tfap2b*, which is expressed in NCCs and their derivatives. Wnt1-Cre-mediated conditional deletion of *Tfap2b* in NCCs resulted in post-natal ocular defects typified by opacity. Histological data revealed that the conditional AP-2β NCC knockout (KO) mutants exhibited dysgenesis of multiple structures in the anterior segment of the eye including defects in the corneal endothelium, corneal stroma, ciliary body and disruption in the iridocorneal angle with adherence of the iris to the cornea. We further show that this phenotype leads to a significant increase in intraocular pressure and a subsequent loss of retinal ganglion cells and optic nerve degeneration, features indicative of glaucoma. Overall, our findings demonstrate that AP-2β is required in the POM for normal development of the anterior segment of the eye and that the AP-2β NCC KO mice might serve as a new and exciting model of ASD and glaucoma that is fully penetrant and with early post-natal onset.

## Abstract (precis)

**Summary:** Tissue-specific deletion of transcription factor AP-2β in the neural-crest-derived periocular mesenchyme generates a novel model of anterior segment dysgenesis and early onset glaucoma in mice.

## INTRODUCTION

Glaucoma is the current leading cause of irreversible blindness worldwide, and due to the rapidly aging population it is predicted to affect over 70.6 million people by 2020 and 111.8 million people by 2040 globally, with a disproportionate prevalence seen in Asian and African countries (Quigley and Broman, 2006; Tham et al., 2014). It is a multifactorial disease process that is characterized by a loss of retinal ganglion cells (RGCs), excavation of the optic nerve head (ONH) and defects of specific visual fields. Elevation in intraocular pressure (IOP) is a major risk factor and, currently, it is the only clinically treatable feature of the disease (Kass et al., 2002). There are many forms of glaucoma, with primary open angle (POAG) being the most prevalent, but angle closure glaucomas are also widespread, particularly in individuals of Asian descent (Quigley and Broman, 2006).

Closed angle glaucomas often arise due to congenital disorders and are classified as anterior segment dysgenesis (ASD) (Ito and Walter, 2014). The anterior segment of the eye – including the cornea, iris, lens, ciliary body and ocular drainage structures such as the trabecular meshwork (TM) and Schlemm's canal – has a complex developmental origin involving cross-talk between the surface ectoderm, optic cup, mesoderm and neural crest (Ito and Walter, 2014). The latter two tissues form the periocular mesenchyme (POM) that eventually gives rise to the mature corneal stroma and endothelium, ciliary body stroma and muscle, anterior iris stroma, iridocorneal angle and drainage structures. In contrast, the lens and corneal epithelium are derived from the surface ectoderm. Although the mechanisms underlying the formation of ASDs are not well understood, many are thought to involve an inappropriate patterning and migration of the POM. Ultimately, failure of normal POM development can lead to anatomical and/or functional defects in the drainage structures responsible for aqueous humor production and outflow. Impediments in aqueous outflow subsequently lead to an increase in IOP, creating an environment favorable to the development of glaucoma (Kupfer and Kaiser-Kupfer, 1978, 1979; Shields, 1987).

Mutations in a number of genes are known to cause human ASD, including *PAX6*, *PITX2*, *PITX3*, *FOXC1*, *FOXE3*, *EYA1*, *CYP1B1*, *LMX1B* and *MAF* (Cvekl and Tamm, 2004; Gage and Zacharias, 2009; Gould et al., 2004; Ito and Walter, 2014; Sowden, 2007). These mutations can result in a spectrum of ocular disorders, which are classified based on the tissues involved (Sowden, 2007). One of these disorders is Axenfeld–Rieger syndrome (ARS) in which patients exhibit abnormalities that are more confined to tissues affecting the angle, as opposed to other ASDs that involve the lens. Features of ARS include adhesions between the iris and cornea, iris hypoplasia, holes in the iris and abnormal chamber angle tissue (Mears et al., 1998; Semina et al., 1996). The most common genetic causes of ARS are mutations in the transcription factors *PITX2* and *FOXC1* (Gould et al., 2004). Both of these regulators are specifically expressed in the developing POM and are thought to be part of a similar regulatory network. Further analysis of these two transcription factors using gene knockout (KO) approaches in mice has demonstrated that the loss of either *Pitx2* or *Foxc1* results in profound failure in anterior segment differentiation, indicating a conserved role in both mouse and human (Gage et al., 1999; Lu et al., 1999). However, these null mice, as well as neural crest cell (NCC)-specific KO mice for either of these two genes, are either embryonic lethal or exhibit such severe eye defects, that it is not feasible to use these models to study anterior segment development beyond birth (Gage et al., 2014, 2005; Kume et al., 1998). A temporal gene KO approach that was employed to delete *Pitx2* expression in mice at a later time point during development did, however, reveal that *Pitx2* is required for maintaining specific lineages within the developing cornea and in preventing angiogenesis (Gage et al., 2014). Despite the importance of *Pitx2* and *Foxc1* for development of the eye, it is clear that other regulatory genes responsible for ASDs remain to be identified.

The AP-2 transcription factor family, consisting of five genes encoding the proteins AP-2α, AP-2β, AP-2γ, AP-2δ and AP-2ε, respectively, is postulated to be a crucial component of the gene regulatory network responsible for the evolution and function of the neural crest in vertebrates (Jin et al., 2015). Moreover, previous studies have demonstrated that members of the AP-2 family, particularly AP-2α and AP-2β, can regulate several aspects of eye development through their function in the surface ectoderm or neural retina (Bassett et al., 2012, 2007, 2010; Dwivedi et al., 2005; Pontoriero et al., 2008; West-Mays et al., 2002, 2003, 1999). Of note, AP-2α and AP-2β function together *in vivo* to control development of horizontal and amacrine cell populations in the embryonic retina and potentially in the post-natal eye (Bassett et al., 2012; Jin et al., 2015). Therefore, we were interested in ascertaining whether the individual AP-2 proteins, or particular combinations of these proteins, might also function in the POM to control eye morphogenesis and function. Here, we focus on AP-2β, encoded by *Tfap2b*, which has not been studied in detail in this tissue. Given that *Tfap2b*-null mice die perinatally (Moser et al., 1997), we utilized Cre-LoxP technology to remove AP-2β specifically from the embryonic neural crest and its derivatives so that we could assess how loss of this gene impacts upon post-natal development of the anterior eye structures. Our findings indicate that AP-2β expression in the POM is crucial for anterior segment development and function and, moreover, that its absence from this tissue generates a valuable new model of human closed angle glaucoma.

## RESULTS

### AP-2β NCC KO mutants exhibit multiple defects in the anterior segment of the eye

**Fig. 1.**
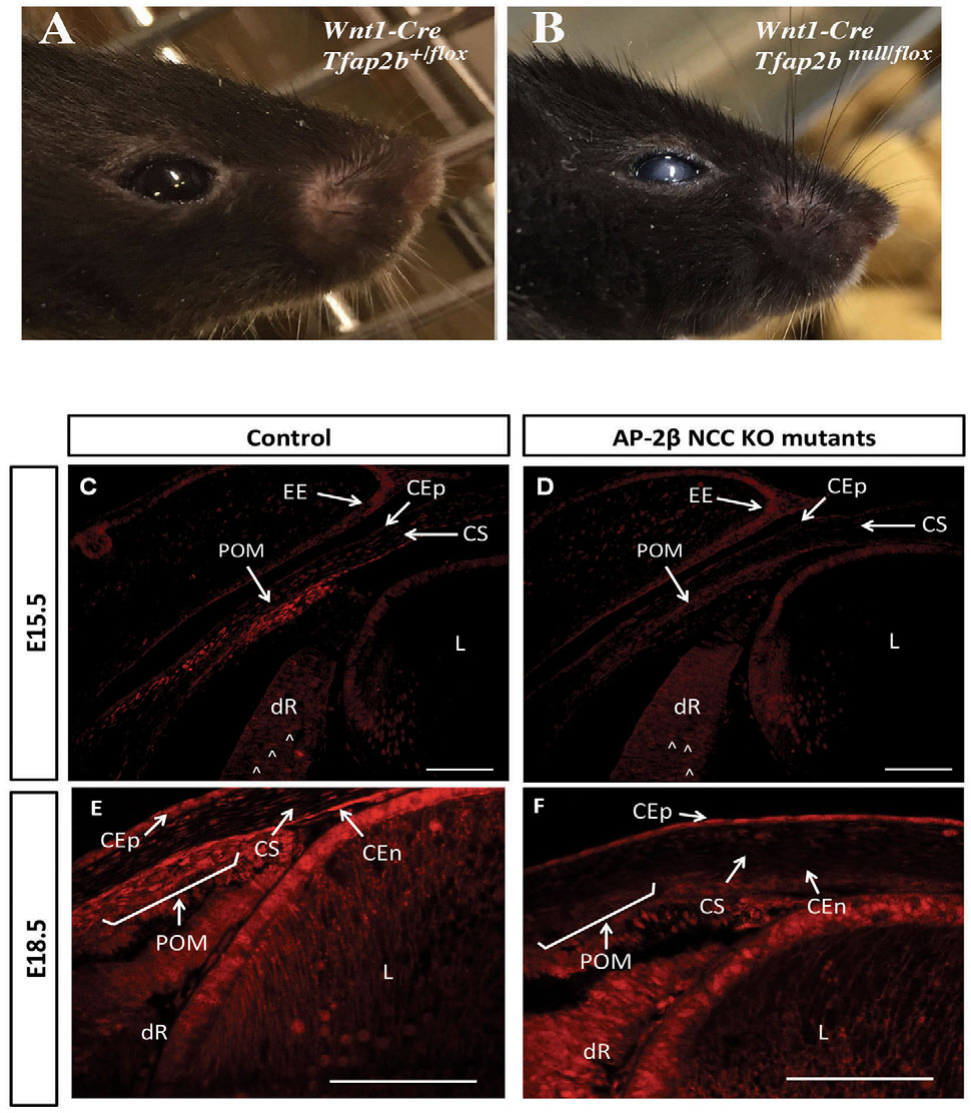
**Image of control and AP-2β NCC KO mutant eye and analysis of AP-2β expression in embryonic eyes.** Image of control (A) and Wnt1-Cre *Tfap2b*-targeted (AP-2β NCC KO) (B) adult mice at 9 months of age. Immunofluorescent detection of AP-2β at E15.5 (C,D) and E18.5 (E,F) in coronal sections of littermate control (C,E) and NCC-specific *Tfap2b*-deleted samples (D,F). CEp, corneal epithelium; CEn, corneal endothelium; CS, corneal stroma; dR, developing retina (arrowheads show expression); EE, eyelid epidermis; L, lens; POM, periocular mesenchyme. Note that AP-2β expression normally seen in the NCC-derived POM and corneal stroma and endothelium is lost in the AP-2β NCC KO mice, but expression is observed in structures including the retina, corneal epithelium and eyelid ectoderm, which are derived from other tissue layers, illustrating the specificity of the gene targeting. Scale bars: 100 μm.

Mice lacking *Tfap2b* die in the early post-natal period, and we sought to determine whether the expression of AP-2β in the neural crest was crucial for viability by specifically targeting a floxed allele of this gene with a *Wnt1-Cre* transgene. Animals lacking *Tfap2b* in the neural crest, hereafter termed AP-2β NCC KO, were viable after birth and initially had a similar appearance to control littermates. However, after eyelid opening, mutant animals were readily identified by the presence of ocular opacity that persisted as the animals aged (Fig. 1A,B). These observations were consistent with previous reports regarding the expression pattern of AP-2β in the NCC-derived POM in mice, which begins by embryonic day (E)9.5 (Bassett et al., 2010) and can be detected in a substantial number of cells populating the developing cornea and angle tissue by E13.5 (Bassett et al., 2012). However, given that expression at later embryonic timepoints has not been examined, we utilized immunohistochemistry to analyze the distribution of AP-2β protein in the anterior segment at E15.5 and E18.5 in both control and mutant samples (Fig. 1C-F). In E15.5 control animals, AP-2β expression was detected in the POM, the developing retina, the corneal stroma, corneal epithelium and eyelid epidermis (Fig. 1C). Subsequently, by E18.5, control embryos also exhibited positive AP-2β staining in the POM, the corneal epithelium, endothelium and stroma as well as the developing retina (Fig. 1E). In contrast, examination of E15.5 and E18.5 AP-2β NCC KO samples demonstrated that expression was absent from the POM, the corneal endothelium and the stroma, which are NCC in origin (Fig. 1D,F). By contrast, the previously reported pattern of AP-2β expression in the developing retina and corneal epithelium (Bassett et al., 2012; Trimarchi et al., 2007; West-Mays et al., 2003), ocular tissues that are not of NCC origin, was retained (Fig. 1D,F). Similarly, the ectodermally derived lens, which showed increased AP-2β expression between E15.5 and E18.5, produced equivalent staining in AP-2β NCC KO samples (Fig. 1; Fig. S1). Taken together, these observations are consistent with a role for AP-2β in development of specific anterior segment structures from the POM.

**Fig. 2.**
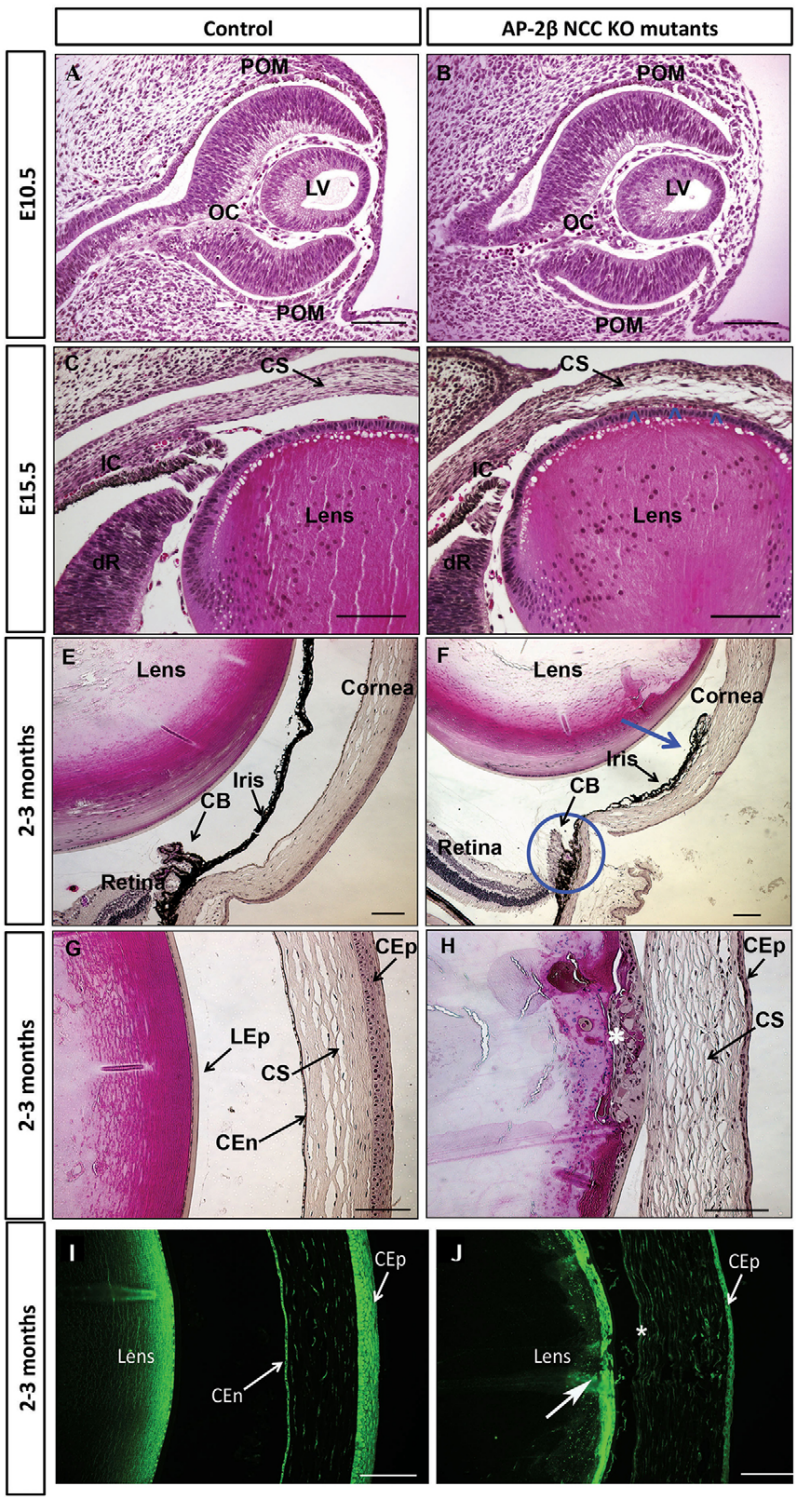
**Aberrant development of the anterior segment in AP-2β NCC KO mutants.** H&E-stained coronal or sagittal sections of E10.5 (A,B), E15.5 (C,D) and 2- to 3-month-old (E-H) control littermates (A,C,E,G) and AP-2β NCC KO samples (B,D,F,H). In D arrowheads indicate the juxtaposition of the cornea and lens compared to control littermates. In F, the blue arrow indicates a closed angle with adherence between the iris and cornea and the blue circle shows hypoplastic ciliary body. In H, the white asterisk indicates the location of the anterior subcapsular cataracts and also note adherence of lens and cornea, as well as thinner corneal epithelium. Immunofluorescent detection of N-cadherin in 2- to 3-month-old sagittal sections of control littermate (I) and an AP-2β NCC KO mutant (J). In J, the asterisk indicates the absence of corneal endothelium staining and the large white arrow points to the subcapsular cataract region of the lens. CB, ciliary body; CEn, corneal endothelium; CEp, corneal epithelium; CS, corneal stroma; dR, developing retina; IC, iridocorneal angle; LEp, lens epithelium; LV, lens vesicle; OC, optic cup; POM, periocular mesenchyme. Scale bars: 100 μm.

Next, to determine the onset and progression of the ocular pathology in AP-2β NCC KO mutants, we performed a detailed histological analysis beginning at E10.5, as this marks the first NCC migration into the presumptive anterior chamber (Gage et al., 2005). The phenotype observed at E10.5, just after the lens vesicle has pinched off from the overlying surface ectoderm and the POM is migrating into the presumptive anterior segment, was remarkably similar for both mutants and control littermates, with no suggestion of abnormal development due to the conditional deletion (*n*=4) (Fig. 2A,B). By contrast at E15.5, once the cornea had formed from the POM and the second wave of mesenchyme had reached the iridocorneal angle, the mutant cornea was less compact, with large gaps within the stroma. Moreover, in stark contrast to the clear separation between the cornea and lens seen in control samples (Fig. 2C), the cornea and lens adhered to one another in the mutant (*n*=2) (Fig. 2D, arrowheads).

At the adult stage (2-3 months old), multiple malformations of the anterior chamber were apparent in AP-2β NCC KO mutant eyes compared to controls (*n*=8). The most striking was the disruption of the iridocorneal angle with the iris adherent to the cornea, creating a fully penetrant closed angle phenotype in the eyes examined (*n*=8) (Fig. 2E,F). The ciliary body was also malformed and potentially rudimentary in the mutants, as it lacked its normally convoluted and lobulated structure (*n*=8) (Fig. 2E,F). Histological analysis in AP-2β NCC KO mice also indicated multiple defects of the corneal layers derived from the POM, including the lack of a clear endothelial layer and a less-cohesive corneal stroma compared with controls (Fig. 2G,H). Pathological changes were also seen in tissues that are not neural crest in origin, including a corneolenticular adhesion and the appearance of anterior subcapsular cataracts, as well as reduced stratification of the corneal epithelial layer (Fig. 2G,H). These latter conditions are presumably secondary defects arising from changes in POM derivatives within the cornea and angle tissue.

**Fig. 3.**
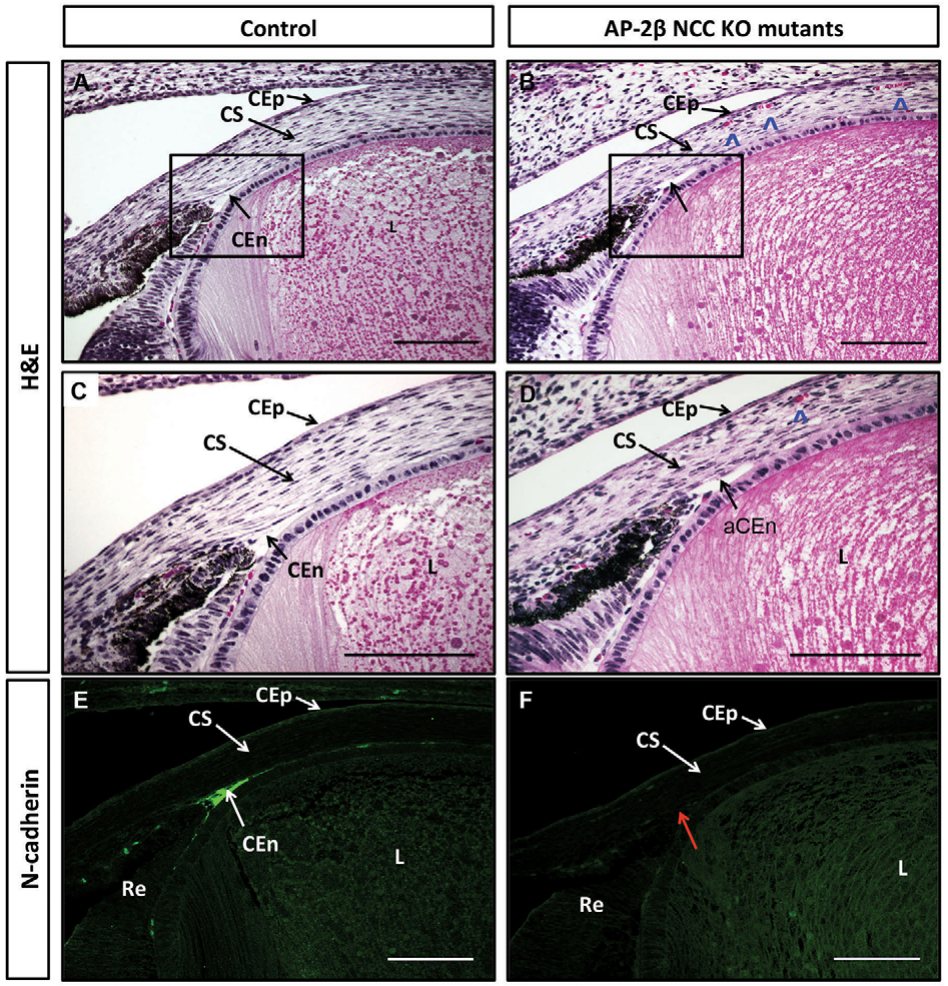
**Embryonic corneal defects in E18.5 AP-2**β **NCC KO mutant embryos.** Coronal sections of E18.5 anterior segments stained using either H&E (A-D) or immunofluorescence for N-cadherin expression (E,F) for controls (A,C,E) or AP-2β NCC KO mutants (B,D,F). Boxed regions in A and B are shown in greater detail in C and D. Red arrow in F shows that corneal endothelium does not stain for endothelial cell marker N-cadherin in absence of *Tfap2b*. Blue arrowheads in B and D indicate presence of red blood cells in mutant corneal stroma. CEn, corneal endothelium; aCEn, absent corneal endothelium; CEp, corneal epithelium; CS, corneal stroma; L, lens; Re, retina. Scale bars: 100 μm.

**Fig. 4.**
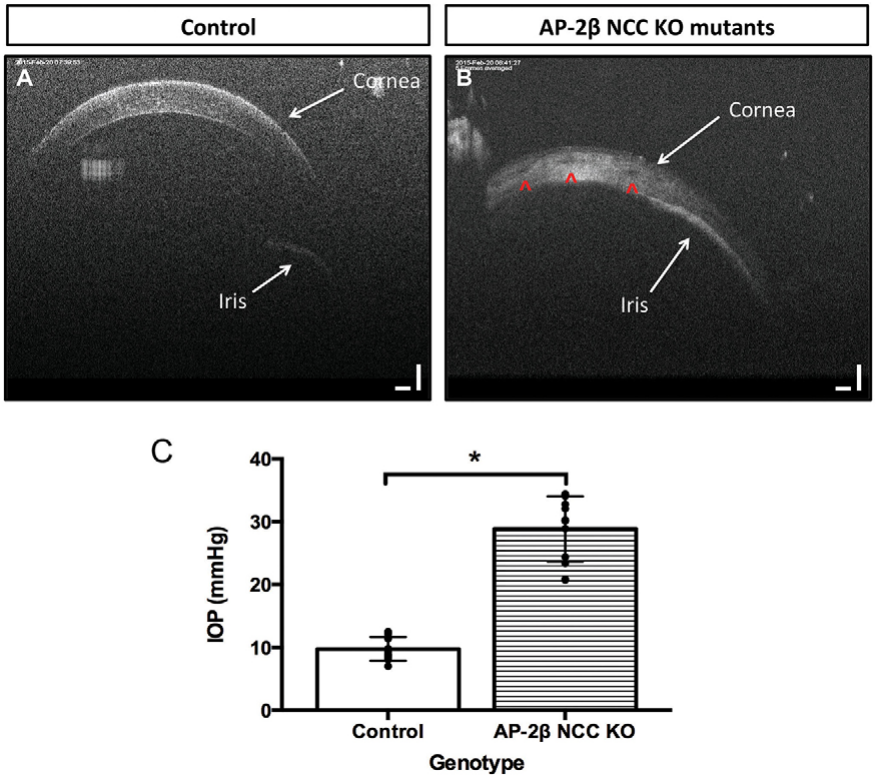
**OCT and IOP analysis of 2-3-month-old AP-2β NCC KO mutants.** OCT images of the anterior chamber of a control littermate (A) and an AP-2β NCC KO mutant (B) at 2 to 3 months of age. White arrows in A and B show relative positions of the cornea and iris, with adherence of the iris to the cornea in B. The presence of round foramina in the corneal stroma of the mutant is shown by red arrowheads (B). (C) A comparison of IOP between 3-month-old AP-2β NCC KO mutants and their control littermates. Individual measurements are overlaid on bar graph comparing IOP between control mice and AP-2β NCC KO mutant mice. Mutant mice have increased IOP (28.87 mmHg±5.19, *n*=12) relative to their control littermates (9.76 mmHg±1.88, *n*=12). Measurements taken with a Rebound Tonometer (TonoLab Vantaa, Finland). Results are mean±s.d. *P<0.0001 (Student's two-tailed *t*-test).

We further examined pathological changes in the adult cornea using immunohistochemistry for the endothelial marker N-cadherin (*n*=8) (Reneker et al., 2000). In control samples, a discrete corneal endothelial layer expressed N-cadherin, but this staining was absent in AP-2β NCC KO mice (Fig. 2I,J). Similarly, N-cadherin staining revealed that the corneal epithelial layer was substantially reduced in thickness in the mutants and that the lens epithelium was disorganized and multi-layered, characteristic of a cataract (Fig. 2J, large white arrow). Next, we used a combination of histology and N-cadherin immunohistochemistry to determine whether abnormalities in the corneal endothelium and angle tissue could be detected prior to birth when AP-2β was specifically removed from the POM (Fig. 3A-F). A comparison of mutant and control littermates (*n*=6) for N-cadherin staining demonstrated that defects in the angle tissue and the corneal endothelium were present at E18.5, showing that these phenotypes resulted from an early developmental defect (Fig. 3E,F). Staining of N-cadherin was also more limited in the mutant corneal epithelium at E18.5, and this was also apparent for AP-2β expression at E18.5, consistent with the reduction of epithelial stratification in the mutant (see Fig. 1E,F and Fig. 2G-J). Histological examination of E18.5 samples further revealed the presence of red blood cells in the corneal stroma of the AP-2β NCC KO mice, but not in the controls (*n*=8; Fig. 3B,D, see arrowheads). Given that the cornea is not normally vascularized, we next examined adult eyes in detail for evidence of blood vessels within this anterior structure by staining for endomucin, a sialomucin specifically expressed by the endothelium of blood vessels. Whereas no endomucin staining was observed in control littermates, clear reactivity was seen in the corneal stroma of AP-2β NCC KO mice at 2 months of age (Fig. S2), and evidence of blood vessels in the cornea could also be seen at the level of gross morphology. Further support for the presence of vessels in the stroma of AP-2β NCC KO mice was obtained using optical coherence tomography (OCT). A comparison between the corneal stroma of the control and mutant mice indicated that, whereas the former had a uniform appearance, the latter showed clear areas indicative of holes or vessels (Fig. 4B, arrowheads). Taken together, these findings provide strong evidence of vascularization of the cornea, indicating a loss of the angiogenic privilege associated with removal of AP-2β from the POM.

### Increased IOP in AP-2β NCC KO mutants relative to control littermates

Obstructions of the aqueous outflow pathway are known to lead to elevations in the IOP in a variety of inducible and spontaneous mouse models of glaucoma (McKinnon et al., 2009). The adhesion between the iris and cornea observed in the post-natal fixed AP-2β NCC KO samples was striking and we, therefore, used OCT to determine whether this defect was also present *in vivo* at 2 months of age. In control mice, OCT showed a clear separation of the iris and cornea whereas AP-2β NCC KO mutants had a consistent adhesion between the cornea and iris, which was detected 360° around the eye (*n*=6) (Fig. 4B). These findings confirm the presence of a severe and fully penetrant closed angle phenotype that develops prior to 2 months of age in AP-2β NCC KO mutants.

In order to determine whether the closed angle phenotype of the AP-2β NCC KO mutants resulted in elevated IOP, rebound tonometry was used on a cohort of AP-2β NCC KO mutants and their control littermates. A significant ∼3-fold elevation of IOP was readily apparent in 3-month-old AP-2β NCC KO mutants (Fig. 4C). The control littermate mice had an IOP measuring 9.76±1.88 mmHg (*n*=12), a value consistent with previous measures of noninvasive IOP readings in 3-month-old C57Bl/6 background strains (Kipfer-Kauer et al., 2010). In contrast, AP-2β NCC KO mutants had a significant elevation of IOP measuring 28.87±5.19 mmHg (*n*=12), a value that is similar to IOP measurements reported in the *nee* mutant mouse model of glaucoma (Mao et al., 2011). These data support the idea that the closed angle phenotype observed in the AP-2β NCC KO mutants results in disruption of the aqueous outflow pathway leading to an increase in IOP. Such an environment is favorable to the development of glaucomatous retinal damage.

### Decreased retinal thickness in AP-2β NCC KO mutants

**Fig. 5.**
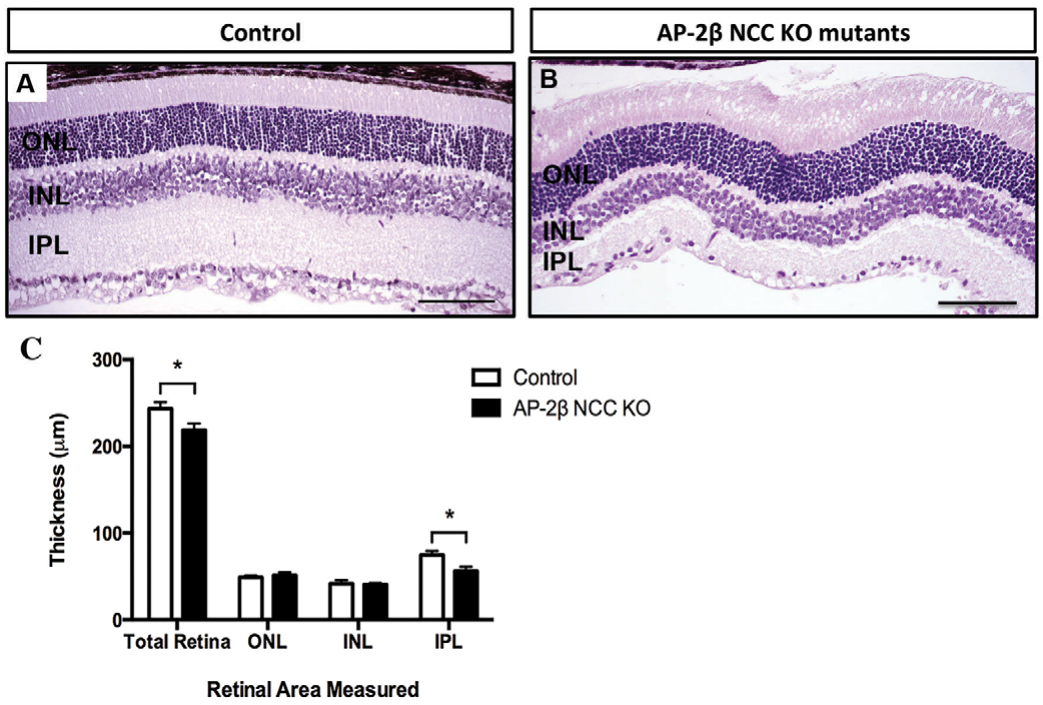
**Decreased overall retinal and IPL thickness in 2- and 3-month-old AP-2β NCC KO mutants.** (A,B) H&E-stained sagittal sections of 2- to 3-month-old control littermate (A) and AP-2β NCC KO mutant (B) retinas. (C) Bar graph comparing average thickness of retinal layers between control and AP-2β NCC KO mutants (*n*=4 each). AP-2β NCC KO mutants at this age have a significantly decreased overall retinal thickness (218.56 μm±7.90, *n*=4) and a decreased inner plexiform layer (IPL) (56.11 μm±5.00, *n*=4) relative to their control littermates (243.47 μm±7.38, *n*=4, and 74.67 μm±4.80, *n*=4, respectively). There was no significant difference in thickness of the outer nuclear layer (ONL) and inner nuclear layer (INL) of the mutants and the controls. Results are mean±s.d. *P<0.005 (Student's two-tailed *t*-test). Scale bars: 100 μm.

A comparison between H&E-stained sections of control and mutant retinae at 2-3 months of age indicated that the overall thickness of this structure was decreased when AP-2β was removed from the neural crest. Measurement of the combined retinal layers revealed that the mutant retinae were significantly thinner relative to that of their age-matched littermates (Fig. 5). Subsequent separate measurements of the outer nuclear layer (ONL), inner nuclear layer (INL) and inner plexiform layer (IPL) indicated that the overall reduction in retinal thickness in the mutants was mainly due to thinning of the IPL, the layer that contains the dendrites of RGCs (Fig. 5). In comparison, the ONL and INL, the layers that contain the nuclei of the photoreceptors and the nuclei of amacrine, bipolar and horizontal cells respectively, exhibited no significant difference in thickness between the mutant and control littermates. This finding suggests that the thinning of the mutant retinae at 2-3 months of age results from a loss of RGCs as the layer that contains their dendrites is significantly reduced.

### Loss of RGCs in 2-month-old AP-2β NCC KO mutant retinae relative to control littermates

**Fig. 6.**
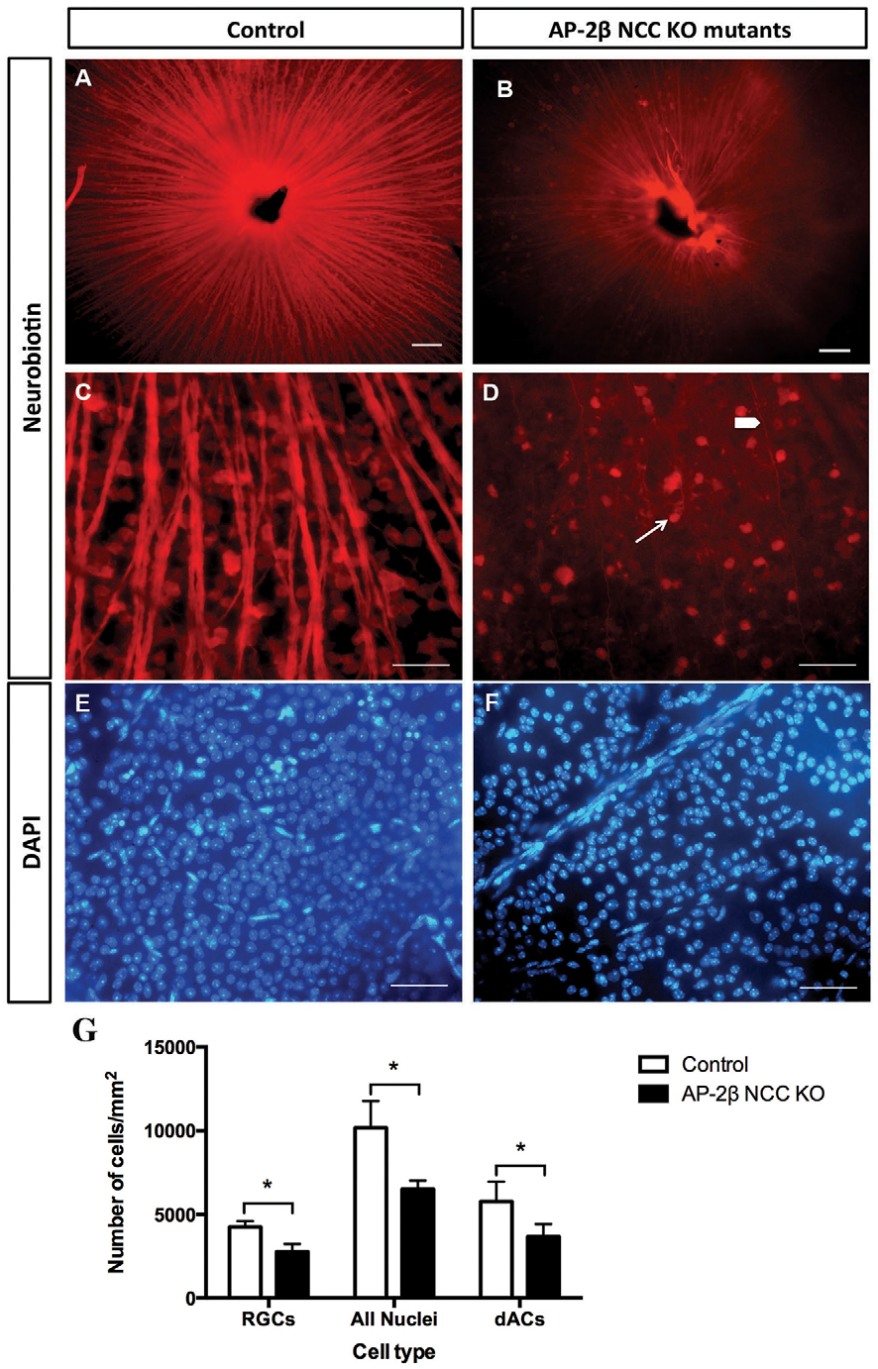
**Decreased number of RGCs, total nuclei and dACs in 2-month-old AP-2β NCC KO mutants.** (A,B) Immunofluorescent frontal view of flat mount retina from control (A) or AP-2β NCC KO mutants (B) following retrograde labeling with neurobiotin. (C,D) Detailed views of images shown in A and B showing reduced numbers of retinal ganglion cell bodies (white arrow) and axons (white arrowhead). (E,F) Flat mount preparation showing the overall decreased number of DAPI-labeled nuclei in the GCL of AP-2β NCC KO mutant retinas as compared to control littermates. (G) AP-2β NCC KO mutants have significantly decreased numbers of RGCs, total nuclei and dACs (displaced amacrine cells) per mm^2^ (2768±461, 6505±511, 3663±755, *n*=3, respectively) relative to their control littermates (4244±365, 10,163±1603, 5774±1178, *n*=3). Results are mean±s.d. *P<0.03 (Student's two-tailed *t*-test). Scale bars: 100 μm.

To determine whether the changes in the anterior segment associated with the increase in IOP in AP-2β NCC KO mutants resulted in a loss of RGCs, the retinal cell type affected in glaucoma, a neuronal tracer method for assessing RGC number was employed (Pang and Wu, 2011). The neuronal tracer Neurobiotin (NB) was placed upon the optic nerve (ON) stump to be taken up and transported retrogradely by the RGC axons to the cell bodies. Fluorescence microscopy of NB-labeled RGCs in retinal whole mounts showed that there was a stark contrast between AP-2β NCC KO mutants and control littermates, with significantly fewer RGCs and their axons in the mutant group (Fig. 6A,B). It was interesting to note that the loss of RGC axons was not uniform, but occurred in a segmental, fan-shaped manner, a phenotype that is similar to what has been observed in the most well-characterized mouse model of glaucoma, the DBA/2J inbred line (Jakobs et al., 2005). Manual counting of DAPI-labeled nuclei and NB-labeled RGC somas was performed and revealed that control littermates had on average 10,163±1603 nuclei/mm^2^ of which 4244±356 nuclei/mm^2^, ∼42%, were from RGCs (mean±s.d., *n*=4 retinae). These values are consistent with total neuronal counts in the ganglion cell layer (GCL) and the calculation that 43% of cells in this layer are RGCs (Jeon et al., 1998). Cell counts of AP-2β NCC KO mutant retinae showed significantly fewer cells, with 6505±511 nuclei/mm^2^ and 2769±462 RGC/mm^2^ (∼43%; *n*=4 retinae) (Fig. 6C,D,G). These data demonstrate that, by two to three months of age, the AP-2β NCC KO mutants exhibited a substantial loss of RGCs. However, the RGCs are only one of the two major cell types in the GCL of the retina, the other being displaced amacrine cells, (dACs). The dACs are interneurons that play an important role in inner retinal visual processing and account for 57% of the cells in the GCL. Therefore, from the previous quantifications of cells in the GCL (above), the number of dACs was calculable by subtracting the number of RGCs from the total number of nuclei present in the GCL. This revealed that the number of dACs in wild-type littermates was 5774±1178 dACs/mm^2^ and that of AP-2β NCC KO mutant retinae was 3641±755 dACs/mm^2^, which is significantly lower (*n*=4 retinae, *P*=0.0225, two-tailed Student's *t*-test). Thus, these data suggest that additional cells within this layer, particularly displaced amacrine cells (dAC), must be lost to a similar extent by 2 months of age in this mouse model (Fig. 6G).

### Loss of retinal ganglion cell axons in ONs

**Fig. 7.**
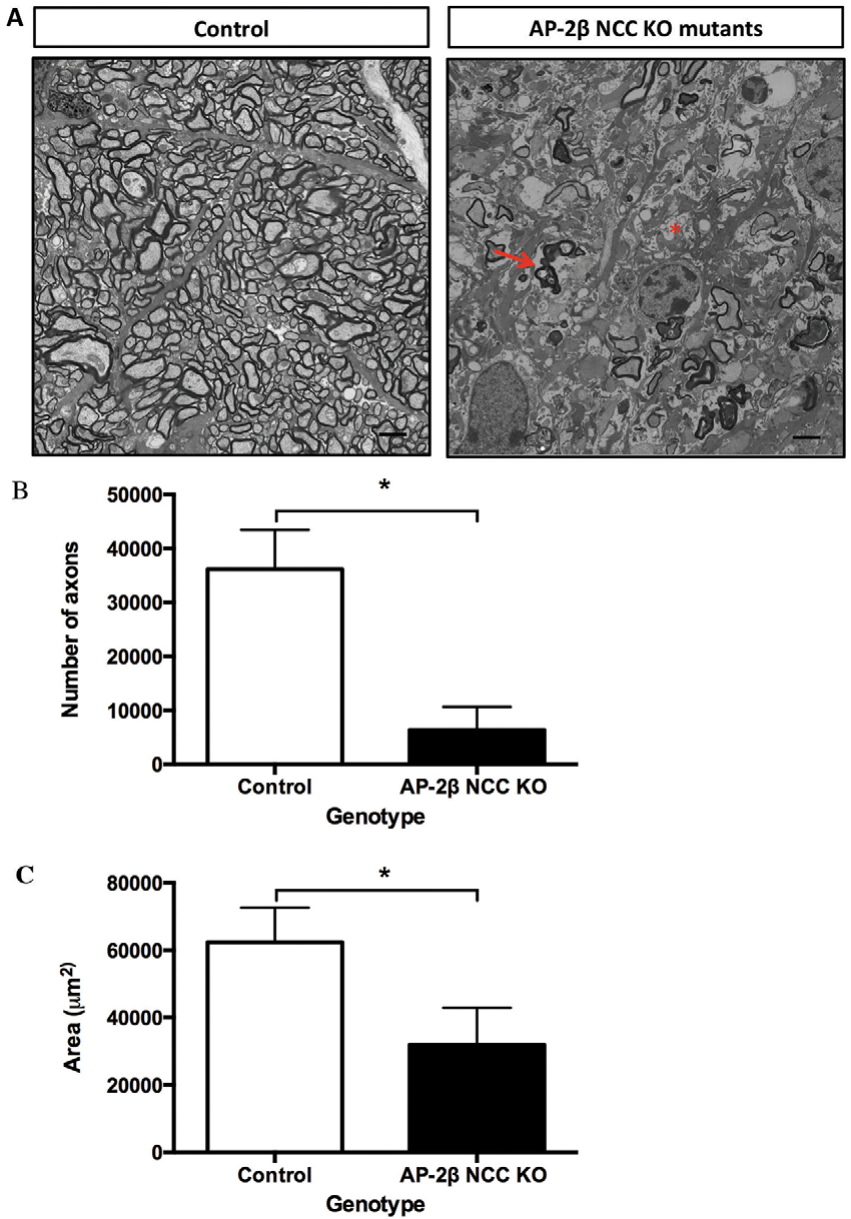
**ON defects in two-month-old AP-2β NCC KO mutants.** (A) Transmission electron micrographs of cross sections of adult ONs from control and AP-2β NCC KO mutants. Degenerating axons (arrow) and areas of severe atrophy (asterisk) are evident. Scale bars: 2 μm. (B) Bar graph comparing axon numbers from cross sections of the ON between control and AP-2β NCC KO mutants. AP-2β NCC KO mutants display a significantly decreased number of myelinated RGC axons (6365±4284, *n*=4) relative to their control littermates (36,143±7276, *n*=4). Results are mean±s.d. *P<0.001 (Student's two-tailed *t*-test). (C) Bar graph comparing ON cross-sectional area between control and AP-2β NCC KO mutants. AP-2β NCC KO mutant ONs have a significantly decreased cross-sectional area (31,871 μm^2^±11,016, *n*=4) when compared to their control littermates (62,286 μm^2^±10,275, *n*=4), supporting the loss of RGC axons within the ON and the development of glaucoma. Results are mean±s.d. *P<0.01 (Student's two-tailed *t*-test).

To test for further evidence of glaucomatous damage in the AP-2β NCC KO model, cross-sections of ONs from 2-month-old mutant and control mice were assessed by electron microscopy to examine the axons of the RGC bodies. The ONs of the control littermates had a normal appearance with many axons of various sizes closely compacted and surrounded by myelin sheaths (Fig. 7A). The ONs of the AP-2β NCC KO mutants, however, had a decreased number of myelinated axons, the presence of degenerating axons (Fig. 7A, arrow) and areas of severe atrophy (Fig. 7A, asterisk). Quantification of the total number of axons also revealed a significant reduction in the number of ON axons in the AP-2β NCC KO mutants when compared to control littermates (Fig. 7B). Presumably, as a consequence of the loss of axons, the cross-sectional area of the mutant ONs was also significantly reduced (Fig. 7C), by ∼2-fold relative to their control littermates, further supporting that the AP-2β NCC KO mutant ONs have undergone severe glaucomatous progression. RGC axon loss in the mutants was also associated with damage of the ON head, consistent with excavation or cupping, further indicative of glaucomatous pathology (Fig. S3).

### Increased glial reactivity of the retina

**Fig. 8.**
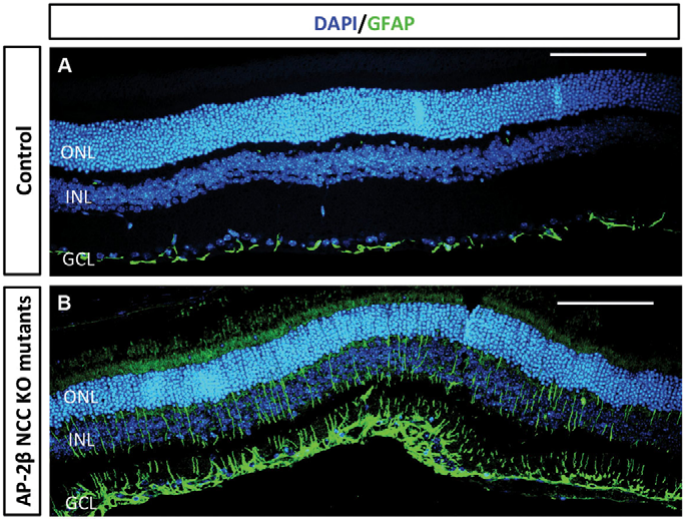
**Increased retinal glial reactivity in the 2-month-old AP-2β NCC KO mutants.** Immunofluorescent detection of GFAP (green) demonstrates increased expression in Müller glia cells in AP-2β NCC KO mutant retinas (B) as compared to control littermates (A) supporting the hypothesis of glaucomatous damage. GCL, ganglion cell layer; INL, inner nuclear layer; ONL, outer nuclear layer. Samples were also stained with DAPI to detect nuclei. Scale bars: 100 μm.

Following central nervous system (CNS) injury and disease, corresponding to the retinal damage in glaucoma, hypertrophy and proliferation of glial cells such as Müller glia and astrocytes will occur, which is also known as gliosis. A hallmark of gliosis is the upregulation of intermediate filaments, such as glial fibrillary acidic protein (GFAP), that are in contact with the cytoskeleton and extracellular matrix and will facilitate the rapid and long-term remodeling of tissue structure. In the control littermates, normal GFAP staining of astrocytes was seen in the GCL and fiber layers extending from the GCL, with no staining present in Müller glia in the other retinal layers as expected (Fig. 8A) (Bjorklund et al., 1985; Sarthy et al., 1991). AP-2β NCC KO mutants, however, exhibited an upregulation in expression of GFAP in the Müller glia (Fig. 8B), similar to what has been previously observed in other animal models of glaucoma (Tanihara et al., 1997; Wang et al., 2000). Taken together, these data indicate that the absence of AP-2β from the POM results in significant changes in the retina and ON that are consistent with glaucomatous pathology.

## DISCUSSION

Glaucoma is a major cause of blindness worldwide and is frequently associated with increased IOP that can damage the retina and ON. There are clear genetic links to human glaucoma, but the underlying mechanisms of disease progression remain unclear, which presents a significant barrier to early detection and treatment. This issue is compounded by the relative lack of animal models that develop glaucoma consistently and with a short latency. The increase in IOP found in glaucoma is often thought to arise from defects in the drainage system of the eye present at the iridocorneal boundary. In open angle glaucoma, these defects are not readily apparent using standard diagnostic or histological approaches; however, in closed angle glaucoma there are clear structural defects associated with the anterior segment that prevent normal drainage of the aqueous humor. In this respect, mutations in *FOXC1*, *PITX2*, *SH3PXD2B* and *LMX1B*, among others*,* cause various ASDs and have a significantly increased risk of early onset glaucoma. *SH3PXD2B*, mutated in glaucoma-associated Frank–Ter Haar syndrome, encodes a cytoskeletal adaptor protein involved in podosome formation that is widely expressed in the eye (Iqbal et al., 2010) although its primary site of action in anterior segment development remains to be determined. In contrast, *FOXC1* and *PITX2*, which are mutated in both Axenfeld–Rieger syndrome and Iridogoniodysgenesis, are transcription factors that function in the NCC-derived POM to regulate development of the anterior segment (Gage et al., 1999, 2005, 2014; Kume et al., 1998; Seo et al., 2012). Lmx1b is an additional transcription factor expressed in the POM and its associated derivatives, which is linked to eye defects in both mouse and human. *Lmx1b*-null mice as well as mice lacking *Lmx1b* in the neural crest have a variety of ocular defects including smaller eyes, hypoplasia of the iris and ciliary body, and the keratocytes of the corneal stroma less densely packed (Jin et al., 2015; Pressman et al., 2000), and conditional mutant mice also exhibit post-natal anterior segment defects. In humans, mutations in LMX1B have been linked to nail-patella syndrome, which has been associated with glaucoma (Knoers et al., 2000; Lichter et al., 1997).

Here, we have identified AP-2β as a further transcription factor in the gene regulatory network involved in development of the anterior segment from the NCC-derived POM. Our previous studies have demonstrated roles for the AP-2 transcription factors in development and differentiation of multiple ocular tissues, including those of the anterior eye segment (Bassett et al., 2012, 2010; Dwivedi et al., 2005; Pontoriero et al., 2008). These studies have focused mainly on *Tfap2a* because homozygous (global) deletion of AP-2α in mice results in a number of ocular phenotypes (West-Mays et al., 1999). In contrast, overt eye defects were not observed in mouse models targeting other members of the AP-2 gene family, including AP-2β (Moser et al., 1997). However, one underlying reason for this is that the *Tfap2b*-null mice die in the perinatal period from presumptive neural transmitter and/or heart defects preventing analysis of the crucial period of anterior segment development that occurs in the weeks immediately after birth (Zhao et al., 2011). Therefore, in this study, we used a new *Tfap2b* floxed allele in combination with the Wnt1-Cre transgene to examine the consequences of loss of AP-2β specifically within the NCC population. Our findings reveal a crucial role for AP-2β in regulating anterior segment development from the POM, and further demonstrate that loss of AP-2β leads to the typical features of primary closed angle glaucoma with increased IOP and significant early post-natal defects impacting upon the retina and ON.

The development of the anterior chamber of the eye has been extensively studied by fate mapping with tissue derivations of each structure being well-defined in both mouse and chick. It is well established that the NCC population, alongside mesoderm, which together comprise the POM, will ultimately contribute to the extraocular muscles, corneal stroma, corneal endothelium, iris stroma, ciliary body muscle, ciliary body stroma and trabecular meshwork of the anterior eye (Gage et al., 2005). Many of these anterior segment structures are directly impacted by the loss of *Tfap2b* in the neural crest, strongly suggesting a cell autonomous role for AP-2β in the regulation of their development. The most striking malformation was the fully penetrant closed angle phenotype resulting from synechia, the abnormal adhesion between the cornea and iris. Potentially, this defect could occur as a direct result of an aberrant interaction between the iris and cornea during development or be due to the additional malformations found in other anterior structures.

The cornea itself performs multiple roles for optimal functioning of the eye, and the AP-2β NCC KO mutants exhibited a variety of corneal phenotypes, affecting all three cell layers of this structure. Specifically, the corneal endothelium was missing, there was disorganization and vascularization of the corneal stroma, and reduced stratification of the corneal epithelium. With respect to the absence of the corneal endothelium, we postulate that this is a direct result of AP-2β removal from the NCC population because this innermost corneal layer is derived from the POM and was also absent in the mutants beginning in embryogenesis. Loss of the corneal endothelium would be expected to impact upon two crucial aspects of corneal function based on previous studies. With respect to the observed synechia, it has been hypothesized that this cell layer provides a barrier between the anterior chamber and the ‘sticky’ extracellular matrix of the corneal stroma, based in part on transgenic mouse models in which the endothelium is lost (Reneker et al., 2000; Waring et al., 1982). Therefore, in the absence of this protective barrier in the AP-2β NCC KO mutants, the iris might have adhered to the exposed extracellular matrix of the corneal stroma resulting in the closed angle phenotype observed. A similar mechanism could account for the observed corneal lenticular adhesions and subsequent subcapsular cataracts that occur in this *Tfap2b* model. A second major function of the corneal endothelium is to regulate hydration and nutrition of the corneal stroma. For example, a mouse model with a tissue-specific knockout of N-cadherin, the major cadherin expressed in the corneal endothelium, exhibited disorganization of collagen fibrils in the stroma, accompanied by corneal edema and clouding (Vassilev et al., 2012). It was hypothesized that the phenotypes in this model were due to the loss of proper regulation of water and macromolecules from exiting and entering the corneal stroma. Given that we also see corneal stromal disorganization and clouding in the *Tfap2b* NCC KO mutants, these stromal phenotypes might be secondary to loss of AP-2β in the endothelial layer. However, we hypothesize that there is also a direct effect of AP-2β in the POM-derived corneal stroma based upon the observed neovascularization that is not seen in other mouse models of endothelial cell loss (Tian et al., 2012). The transparency on the cornea is crucial for optimal optical performance, and its maintenance of avascularity, recently termed angiogenic privilege, is a necessity for proper visual function (Azar, 2006). Our data indicate that AP-2β expression in the NCC population is required in order to establish angiogenic privilege during the development of the cornea.

Reduced stratification of the corneal epithelium, from five- or six-cell layers to only two, was also observed in the AP-2β NCC KO mutants. Reduced corneal epithelial stratification has also been observed in other mouse models lacking a corneal endothelium (Reneker et al., 2000; Vassilev et al., 2012). Interestingly, in some studies, the corneal epithelium is seen to return to a five- or six-cell layer epithelium in areas of the cornea where the endothelium remained present (Reneker et al., 2000). Given that the Wnt1-Cre transgene used in the current study did not target the corneal epithelium, the reduced stratification observed is presumed to have been a secondary effect of loss of AP-2β in the corneal endothelium. In this context, we have also examined the anterior segment of *Tfap2b*-null mice just prior to birth to determine whether additional corneal defects were apparent at the level of morphology (Chen et al., 2016). These studies indicate that the *Tfap2b*-null and *Tfap2b*-NCC knockout mice have very similar phenotypes at E18.5, with no additional overt pathology observed in other regions of the anterior segment, including the corneal epithelium. However, it was not possible to examine later defects in the *Tfap2b*-null mice, including features of glaucoma, due to perinatal lethality. These findings strongly suggest that it is the action of AP-2β in the NCC component of the POM that is responsible for the role of *Tfap2b* in anterior segment development, but further study using additional Cre recombinase transgenic lines will be needed to determine the exact cell autonomy of the defects observed in the various corneal layers. Similarly, whether the closed angle phenotype is solely a consequence of the absent endothelium, or is due to other anomalies related to tissues within the anterior angle, or even to loss of *Tfap2b* earlier in the POM itself, remains to be determined.

In combination, the results from the characterization of the AP-2β NCC KO model place this transcription factor in a similar regulatory network for anterior segment development as *Foxc1* and *Pitx2.* Mouse mutants that have a conditional deletion of Foxc1 or Pitx2 in the POM or its derivatives also develop neovascularization of the cornea (Gage et al., 2014; Seo et al., 2012). In addition, these mouse models exhibit similar anterior segment phenotypes as the new AP-2β NCC KO mutants including lack of formation of the corneal endothelium, disorganization of the corneal stroma and abnormalities of the corneal epithelium (Gage et al., 1999; Kidson et al., 1999). The possibility that these three transcription factors might function in the same gene regulatory network for development of the anterior segment has prompted an analysis of the interplay between these genes. Initial studies have focused on Pitx2 and AP-2β, and have indicated that AP-2β levels are significantly downregulated in the absence of Pitx2 in the POM (Chen et al., 2016). Further studies will be needed to determine how AP-2β levels are affected in the absence of Foxc1 and how these three genes interact to direct development of the anterior segment. Mutations involving Foxc1 and Pitx2 not only cause eye defects in mice, but are also responsible for several human eye defects, including Axenfeld–Rieger syndrome. As yet, there is no substantial evidence linking *TFAP2B* to human eye disease; however, this gene is mutated in Char syndrome, a rare dominantly inherited condition that is associated with patent ductus arteriosus, facial dysmorphology and digit defects as well as sleep disorders (Gelb and Chung, 2014; Mani et al., 2005). Although reports of eye examination are uncommon in Char syndrome patients, it should be noted that several individuals do present with strabismus, and this could reflect a function of AP-2β in the NCC-component of the extraocular muscles. In future, it would be potentially important to include a detailed examination of the eye in Char syndrome patients. Alternatively, given that the mouse model represents a *Tfap2b* loss of function, whereas Char syndrome is inherited in a dominant manner suggestive of *TFAP2B* haploinsufficiency, these specific genetic alterations might result in a different spectrum of eye pathology.

We have further characterized the post-natal defects in AP-2β NCC KO and shown that the presence of ASDs is accompanied by the hallmarks of congenital closed angle glaucoma. First, the AP-2β NCC KO mutants exhibited significantly higher IOP levels than their control littermates. This presumably results from the closed angle phenotype that blocked access to both the trabecular meshwork and Schlemm's canal as well as the uveoscleral pathway. Rebound tonometry was used in this study to measure IOP and this non-invasive method is reliant upon the characteristics of the cornea to calculate the IOP. Thus, it is possible that the corneal phenotype present in these mutants in some way impacted upon the measurements recorded, although we do not favor this hypothesis given the highly penetrant closed angle phenotype. Along with the raised IOP, there was also a loss of RGCs as early as six weeks of age, with a significant loss calculated at two months of age, as well as ON cupping. Retinal defects were apparent through thinning of the IPL, retrograde labeling, and ON counts and cross-sectional area measurements. It should also be reiterated that the fan-shaped sectorial loss of RGCs observed in the mutants is consistent with damage to bundles of axons at the ONH as a result of increased IOP, creating a pattern of loss that is similar to that found in the human condition of glaucoma (Jakobs et al., 2005).

Mouse models of glaucoma have proven to be extremely useful in not only helping to determine the molecular and genetic interactions that occur during the disease process but also in providing an avenue for testing future therapeutic treatments to arrest and potentially reverse retinal damage. A variety of mouse models of glaucoma exist, including for primary closed angle, secondary closed angle, primary open angle and normal tension. The AP-2β NCC KO model represents a new fully penetrant congenital primary closed angle model. Of the few spontaneous or genetic mouse models that are used, such as the well-established DBA/2J inbred strain, our model provides some advantages. The first is the timeline at which our mutants begin to lose RGCs. Spontaneous models such as the widely used DBA/2J inbred strain, and myocilin and α1 subunit of collagen type I transgenic models do not begin to experience loss of RGCs until 6-12 months of age with only a modest elevation in IOP (Aihara et al., 2003; Libby et al., 2005; Senatorov et al., 2006), whereas in our model these changes occur more quickly. Second, the AP-2β NCC KO mutants also exhibited these glaucomatous features in a fully penetrant manner whereas the DBA/2J inbred strain has highly variable phenotypes with varying loss of RGCs therefore necessitating a large cohort of animals to be examined in order to gain significant data (Libby et al., 2005). In this context, the phenotype of the AP-2β NCC KO mutants more closely mimics that of the genetic *Sh3pxd2b* (*nee*) mouse model, which exhibit a considerable increase of IOP and early loss of RGCs (Mao et al., 2011). This likely has to do with the similarity in the closed angle phenotype exhibited in each of these models.

Glaucoma is typically diagnosed based on observations of excavation of the ONH and thinning of the nerve fiber layers that house the nuclei and dendrites of the RGCs, respectively (Moura et al., 2012), features we see in the AP-2β NCC KO model. The thinning of these layers indicates RGC loss, whereas the remaining layers of the retina typically retain their thickness. It was initially thought that only RGCs were damaged in glaucoma. However, several recent studies in humans and animal models have suggested that amacrine cells might also be lost (Cooper et al., 2008). Thus, future studies to understand the progressive loss of the different retinal cell types in our model will be important in understanding how IOP influences retinal degeneration and how this compares to the human condition of glaucoma.

In summary, the current study demonstrates that AP-2β has a crucial autonomous function in the NCC-derived POM for development of the cornea and angle tissue. Previous experiments have also shown that AP-2β can act in the retina in conjunction with AP-2α to regulate the development of specific neuronal cell types (Bassett et al., 2012). Therefore, it is now apparent that *Tfap2b* has an extensive influence on multiple aspects of eye development, serving as an important component of the gene regulatory network responsible for anterior segment formation. Finally, because the AP-2β NCC KO mice exhibit a significant elevation in IOP and a subsequent loss of RGCs accompanied by ON degeneration, they might serve as a valuable new model of human closed angle glaucoma.

## MATERIALS AND METHODS

### Generation of AP-2β NCC KO mutants

All animal procedures were performed in accordance with the ARVO Statement for the Use of Animals in Ophthalmic and Vision Research. Conditional deletion of *Tfap2b* from NCCs was achieved by breeding *Wnt1-Cre* mice heterozygous for a *Tfap2b*-null allele (Moser et al., 1997; Danielian et al., 1998) to homozygous floxed *Tfap2b* mice (T.W., unpublished). Genotyping for *Tfap2b* null, *Tfap2b* flox and *Wnt1-Cre* alleles was performed by PCR using DNA extracted from ear clippings using the EZNA Tissue DNA Kit (Omega Bio-Tek) with relevant primer sequences shown in Table S1. Mice that were *Wnt1-Cre Tfap2b^null/flox^*, hereafter termed AP-2β NCC KO animals, were gender matched to control littermates that still contained at least one functional copy of *Tfap2b* in the NCC.

### Histology and immunofluorescence

Whole eyes of the AP-2β NCC KO mutants were fixed in 10% neutral buffered formalin for 24 h and then stored in 70% ethanol. Samples were processed (Histology Department, McMaster University) and embedded in paraffin (Paraplast Tissue Embedding Media, Fisher Scientific). Serial sections were cut to 4 μm in thickness and used for immunofluorescent analysis or hematoxylin and eosin (H&E) staining. Paraffin-embedded sections were deparaffinized in xylene, rehydrated through 100%, 95% and 70% ethanol, followed by water, treated with 10 mM sodium citrate buffer (pH 6.0; boiling for 20 min) for antigen retrieval, blocked with normal serum for 1 h and incubated with primary antibodies overnight at 4°C. Indirect immunofluorescence was performed using the following primary antibodies: 1:50 rabbit anti-AP-2β (2509S, Cell Signaling), 1:100 mouse anti-N-cadherin (610920, BD Transduction) and 1:100 rat anti-endomucin (14-5851, eBioscience). Fluorescent secondary antibodies, which were conjugated to either Alexa Fluor 488 or 568, and of appropriate specificity for the primary antibody species (1:200; A11029 and A11011, Invitrogen, Molecular Probes) were incubated for 1 h at room temperature. All stains were mounted with ProLong Gold antifade reagent containing 4,6-diamino-2-phenylindole (DAPI) (Invitrogen, Molecular Probes). Staining was visualized with a microscope equipped with an immunofluorescence attachment (Leica), and images were captured with a high-resolution camera and associated software (Open-Lab; Improvision, Lexington, MA). Images were reproduced for presentation with image-management software (Photoshop 7.0; Adobe Systems Inc.). Retinal thickness measurements were taken from H&E-stained slides of mice at 2-3 months of age using ImageJ (NIH) for quantification.

### Anterior segment imaging with the Phoenix Micron IV imaging microscope and OCT attachment

AP-2β NCC KO mutants and their control littermates were weighed and anesthetized with an intraperitoneal (i.p.) injection of 2.5% avertin at 0.015 ml/g body weight. Whiskers were trimmed to ensure no obstruction of the imaging system and two sets of eye drops applied in order to dilate the pupils (0.5% Tropicamide Ophthalmic Solution and 2.5% Phenylephrine Hydrochloride Ophthalmic Solution, AKORN, Lake Forest, IL). The corneas were maintained well moistened throughout the procedure with consistent application of Tear-Gel ophthalmic liquid gel (Alcon Canada, Mississauga, ON, Canada) to prevent drying. The Phoenix Micron IV rodent eye imaging system, with optical coherence tomography (OCT) attachment (Phoenix Research Labs, Pleasanton, CA), was utilized to image the corneas and anterior chambers of the AP-2β NCC KO mutants and their control littermates.

### IOP measurements

Three-month-old AP-2β NCC KO mutants and their control littermates were weighed and anesthetized with an i.p. injection of 2.5% avertin at 0.015 ml/g body weight. LACRI-LUBE (Allergan Inc., Markham, ON, Canada) was applied to the eyes to maintain a moist tear film and whiskers were trimmed so there was no impediment of the probe. A validated commercial rebound tonometer (TonoLab, Vantaa, Finland) was mounted on a retort stand with the probe aligned horizontally and perpendicularly to the central cornea, with the tip positioned at 2-3 mm from the eye as previously advised (Chatterjee et al., 2013). A mean of six measurements was determined for each eye and this was repeated ten times in order to calculate an overall mean. Measurements were only accepted if they registered as TonoLab readings with the best reproducibility (no bar symbol) or the next best reproducibility (bar present at the bottom of the screen) as per the manufacturer's manual. All measurements were taken between 13:00 to 15:00 in the afternoon and 3 min after injection of avertin, as this has been previously shown to be a period of stable IOP (Ding et al., 2011).

### RGC tracing with Neurobiotin

Animals were deeply anesthetized and the eyes enucleated before animals were euthanized. The ONs were first removed from each eye to be processed separately for electron microscopy (EM). The eyes were then placed cornea down in a moist, warm, oxygenated chamber. A piece of gelfoam soaked in 8% Neurobiotin (NB) (Vector Laboratories, Inc.) was placed on the optic stump. The eyes were incubated in the chamber for 1 h, after which they were fixed for 2 h in 4% paraformaldehyde. After washes, the retinae were removed and incubated in Texas-Red-conjugated Streptavidin (Jackson ImmunoResearch Laboratories) overnight at room temperature. The next morning the retinae were washed and mounted onto slides with Vectashield containing DAPI (Vector Laboratories). All staining was visualized with a microscope equipped with an immunofluorescence attachment (Zeiss), and images were captured with a high-resolution camera and associated software (Zen; Zeiss). Images were reproduced with image-management software (Photoshop 7.0; Adobe Systems Inc.). Retinal whole mounts with DAPI-labeled nuclei and NB-labeled RGCs were sampled four times in the mid-periphery of each petal; the counts of the nuclei were tallied and averaged to be considered as one sample. Quantification of the nuclei of RGCs was performed using the manual ‘Cell counter’ plug-in with quantification software ImageJ (NIH) in order to ensure each soma was counted only once and an automatic tally was generated.

### Electron microscopy

Following careful dissection of the ONs from the ocular orbit, they were immediately immersed in a solution of 4% PFA and 2% glutaraldehyde for 2 h. ONs were then washed in phosphate buffer solution and post fixed in a 1:1 solution of 2% aqueous OsO_4_ and 0.1 M cacodylate buffer for 1 h at room temperature. Serial dehydration occurred in acetone (20%, 50%, 75%, 90% and 100%), and samples were embedded in EPON resin (Canemco and Marivac, QC, Canada). Thin sections of ONs were created using a diamond knife with an ultramicrotome (Reichert-Jung Ultracut E Microtome; American Instruments) and collected on 200 mesh copper grids (Electron Microscopy Sciences, McMaster University). Sections were stained with uranyl acetate and Reynolds' lead citrate and viewed using a JEOL 1200EX electron microscope at an accelerating voltage of 80 kV. Images were collected and stored for later analysis and quantification. Five regions of each ON section were sampled in order to determine axon numbers. The axon numbers were then averaged and considered one sample.

### Statistical analyses

Comparisons of IOP measurements, retinal thickness, DAPI and RGC counts on retinal whole mounts and ON samplings between AP-2β NCC KO mutants and control littermates were tested for significance with a Student's two-tailed *t*-test (Prism 6, GraphPad Software). Data were considered significant when *P*<0.05. All values are expressed as mean±s.d.
